# Photochemical degradation of trypan blue

**DOI:** 10.1371/journal.pone.0195849

**Published:** 2018-04-10

**Authors:** Tobias Brockmann, Véronique Blanchard, Philipp Heretsch, Claudia Brockmann, Eckart Bertelmann

**Affiliations:** 1 Berlin Institute of Health (BIH), Berlin, Germany; 2 Charité – Universitätsmedizin Berlin, corporate member of Freie Universität Berlin, Humboldt-Universität Berlin, and Berlin Institute of Health, Institute of Laboratory Medicine, Clinical Chemistry and Pathobiochemistry, Berlin, Germany; 3 Freie Universität Berlin, Institute of Chemistry and Biochemistry, Berlin, Germany; 4 Charité – Universitätsmedizin Berlin, corporate member of Freie Universität Berlin, Humboldt-Universität Berlin, and Berlin Institute of Health, Department of Ophthalmology, Berlin, Germany; Massachusetts General Hospital, UNITED STATES

## Abstract

**Purpose:**

To investigate the photochemical degradation of trypan blue (TB) and to identify decomposition products.

**Methods:**

Defined solution samples of TB and a mixture with lutein/zeaxanthin were exposed to blue light. Thermal degradation processes were ruled out using controls not subjected to irradiation. All samples were analyzed using optical microscopy, UV/Vis spectroscopy, matrix-assisted laser desorption/ionization time-of-flight (MALDI-TOF) mass spectrometry and nuclear magnetic resonance (NMR) spectrometry. Degradation kinetics were determined based on changes in absorbance; intermediates were identified by analyzing mass differences of characteristic fragment ion peaks within the fragmentation patterns, and assignments were verified by NMR.

**Results:**

TB demonstrated a photochemical degradation, which can be triggered by lutein/zeaxanthin. Intermediates vary depending on the presence of lutein/zeaxanthin. The self-sensitized photodegradation of TB occurs under generation of dimethyl sulfate and presumed formation of phenol. In contrast, within the presence of lutein/zeaxanthin the decomposition of TB indicates the formation of methoxyamine and sulfonyl arin. Thermal degradation processes were not observed.

**Conclusions:**

TB demonstrated a photodegradation that may be triggered by lutein/zeaxanthin and results in the formation of cytotoxic decomposition products. Our findings contribute to understand degradation mechanisms of TB and may elucidate previous clinical and experimental observations of cellular toxicity after TB application.

## Introduction

Trypan blue is a water-soluble diazo dye that was introduced in 1904 by Paul Ehrlich for the treatment of African trypanosomiasis (sleeping sickness).[1] Decades later—in 1967—the vital dye was first applied in ophthalmology for diagnostic purposes to stain the cornea and conjunctiva.[2] Since then, trypan blue dyeing has become an integral method in intraocular surgery of the anterior and posterior segment. It is used to stain and visualize intraocular membranes to facilitate their intraoperative handling and removal during capsulorrhexis,[3] descemetorrhexis,[4, 5] and peeling of the internal limiting membrane or epiretinal membranes.[6, 7] Due to its common intraocular application, the dye has become subject of clinical and experimental investigations to determine its safety profile. The safety of an intraocular application is being discussed controversially.[8–12] Dose, exposure time as well as light dependent toxicities on the retinal pigment epithelium and the neurosensory retina have been matter of discussion in several in vitro, in vivo[13, 14] and ex vivo[15–19] studies. Indications of toxicity were supported by clinical observations of retinal damage after trypan blue application.[20–23] In summary, previous investigations on trypan blue toxicity revealed two substantial conclusions: First, the toxicity profiles are highly variable comparing the application within the anterior[9] and posterior ocular segment;[12, 18] and second, a light-dependent toxicity was found.[24]

In this account, an interaction of trypan blue with photodegradable antioxidants, such as lutein and zeaxanthin seems obvious. These compounds are highly concentrated within the retina, but almost absent within tissues of the anterior segment.[25] The aim of the present in vitro study was to examine the photoreactivity of trypan blue and to investigate interactions with lutein/zeaxanthin. Photodynamic processes were quantified using UV/Vis spectroscopy and degradation metabolites were assessed qualitatively by matrix-assisted laser desorption/ionization time-of-flight (MALDI-TOF) mass spectrometry and nuclear magnetic resonance (NMR) spectrometry.

## Materials and methods

### Reagents and irradiation

Commercially available pharmaceutical solutions of 0.5 mg/ml trypan blue (RS-Blue^®^; Alchimia, Padova, Italy) and a solution mixture of 0.4 mg/ml trypan blue with 10.0 mg/ml lutein/zeaxanthin (Phacodyne^™^; Kemin, Des Moines, USA) were used. While lutein and zeaxanthin are poorly soluble in water, the purchased substances contain specially treated “water-soluble lutein”, as disclosed by Sousa-Martins.[26] Twenty-microliter aliquots were filled into borosilicate capillaries (#9201015; Hirschmann Laborgeräte GmbH, Eberstadt, Germany) and sealed to avoid evaporation. Samples were irradiated with blue fluorescent lamps at an intensity of 3.0 mW/cm^2^ at a wavelength of 460 nm (Bili-Compact; Weyer GmbH, Kürten-Herweg, Germany). Taking into account the transmittance rate of 90% of borosilicate glass at 460 nm, an effective irradiation intensity of 2.7 mW/cm^2^ was applied. Regarding intraoperative illuminations, the irradiation intensities of operating microscopes vary between 100 and 1000 mW/cm^2^[27, 28] and comparisons of endoillumination systems disclose intensities between 23 and 285 mW/cm^2^ within a 10-mm retina distance.[29] In this context, even sunlight is defined with an irradiation as equal to 100 mW/cm^2^ (IEC 904–3:1989). Therefore, we considered a minimal intensity of 2.7 mW/cm^2^ for the proportion of short-wave light (≤ 460 nm wavelength) as appropriate for our investigations.

With regard to the intraocular oxygen pressure, which is rather hypoxic at pO_2_ ~13 mmHg compared to the arterial oxygen pressure of pO_2_ 75–100 mmHg,[30] the borosilicate capillaries were not just sealed to avoid evaporation but also to attain an oxygen exhaustion that comes close to possible intraocular conditions.

While, the aim of this in vitro study was not to fully imitate intraoperative conditions, but to reveal fundamental degradation mechanisms and to investigate gradual and delayed light-induced constitutional changes, irradiation times ranged from 0.5 to 120 hours (0.5, 1, 2, 3, 6, 9, 12, 15, 18, 24, 30, 36, 42, 48, 72, 96 and 120 h). Samples were stored at 4°C and protected from light between the predefined irradiation intervals. Two samples not subjected to irradiation, one stored at 4°C and another stored at room temperature, served as controls. Samples were subsequently subjected to optical microscopy, photo spectroscopy, mass spectrometry and NMR spectrometry. For NMR spectrometry, 0.7 ml samples of trypan blue solution or the solution mixture of trypan blue and lutein/zeaxanthin were analyzed before and after 120 hours of irradiation.

### Optical microscopy

A drop (~50 μl) of the analytes was placed on microscope slides, which were then sealed with coverslips. Mounted slides were examined using the Axio Imager.M2 optical microscope (Zeiss, Jena, Germany) and analyzed using ImageJ version 1.41 (NIH; Bethesda, USA; http://rsb.info.nih.gov/ij/).

### UV/Vis spectroscopy

All samples were measured in triplicate within a bandwidth of 220–750 nm using an UV/Vis spectrophotometer (NanoDrop 1000; Thermo Fisher Scientific, Wilmington, USA). Spectroscopic values were digitized by NanoDrop 1000 software version 3.8.0 and analyzed with SPSS version 24.0 (IBM, Armonk, USA). The obtained spectroscopic results were evaluated in accordance with Lambert-Beer’s law (E_λ_ = ε_λ_ ⋅ c ⋅ d; E_λ_ = absorbance, ε_λ_ = molar absorptivity, c = concentration and d = thickness of the measuring cell). Molar absorptivity and thickness (of 1 mm) were treated constants, so the concentrations remained as influencing variable.

### Mass spectrometry

MALDI-TOF mass spectra were recorded in a mass range of 200–5000 Da on an Ultraflex III mass spectrometer (Bruker Daltonics, Bremen, Germany) equipped with a Smartbeam laser and a LIFT-MS/MS facility. Each spectrum resulted from 3000 laser shots. The system was calibrated using a dextran ladder, as shown in S1 Fig. The trypan blue—lutein/zeaxanthin solutions (0.5 μl) were mixed on the ground steel target in a 1:1 ratio with the matrix consisting of 2,5-dihydroxybenzoic acid (10 mg/ml) dissolved in 10% aqueous acetonitrile. Baseline corrections and peak picking were performed using FlexAnalysis software version 3.0.9 (Bruker Daltonics, Bremen, Germany).

### Nuclear magnetic resonance spectrometry

NMR spectra were recorded on a Bruker AVANCE III 700 MHz instrument (Bruker Biospin, Rheinstetten, Germany) using 32 scans for ^1^H NMR and calibrated against residual incompletely deuterated solvent [*δ* = 2.50 ppm (parts per million), DMSO-*d*_5_ (for DMSO-*d*_6_)] as an internal reference at 298 K. The following abbreviations were used to explain the multiplicities: s = singlet, d = doublet, and m = multiplet. Preparation of the samples included removal of all volatile materials under vacuum (0.001 mbar at 25 °C), filtration, and re-dissolution in DMSO-d_6_. Several other deuterated solvents had been tested for their ability to completely dissolve the samples, among them CDCl_3_, CD_2_Cl_2_, C_6_D_6_, acetone-*d*_6_, and MeCN-*d*_3_, but all proved to be inferior to DMSO-*d*_6_.

### Statistical analysis

Normally distributed variables with equal variances were compared using the Student t-test. Variables with unequal variances were compared using the Welch-test for independent samples. Pearson’s correlation coefficient was applied for normally distributed variables; otherwise Spearman’s correlation coefficient was used. A p value less than 0.05 was considered statistically significant.

## Results

### Photodynamic degradation

**Fig 1:**
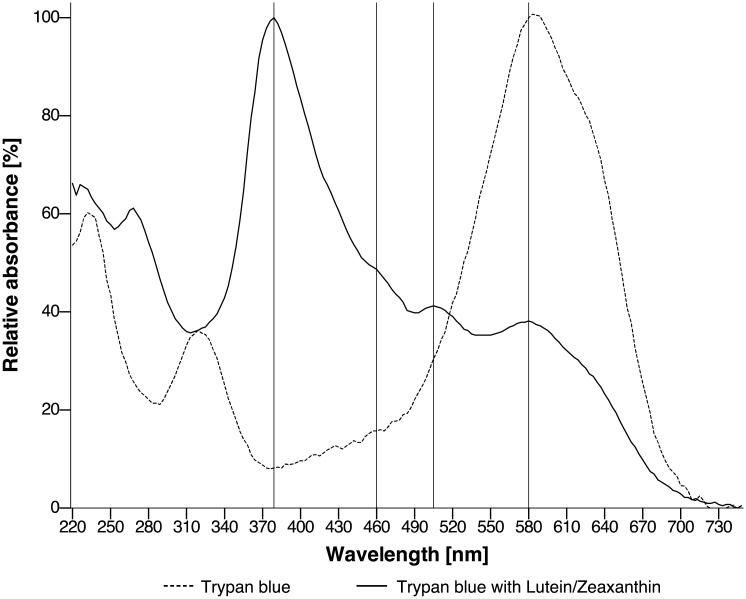
Photometric spectra of trypan blue (dotted line) and trypan blue combined with lutein/zeaxanthin (black line) in aqueous solution. Characteristic absorption maxima are highlighted with marking lines: 379 nm (lutein/zeaxanthin), 580 nm (trypan blue) and 460 nm/505 nm (lutein/zeaxanthin diacetate).

UV/Vis spectroscopy within a bandwidth of 220–750 nm revealed substance-specific absorption spectra of trypan blue and lutein/zeaxanthin, as shown in Fig 1. Trypan blue demonstrated a major 580 nm and a minor 325 nm band. Lutein/zeaxanthin, in its particular formulation,[26] demonstrated a major 379 nm band and two minor bands of lutein/zeaxanthin diacetate at 460 nm and 505 nm, respectively.[31, 32]

**Fig 2:**
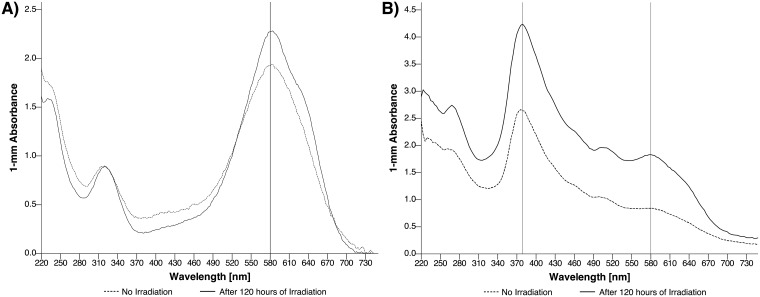
Photometric spectra of trypan blue (a) and trypan blue combined with lutein/zeaxanthin (b) in aqueous solution with no (black line) and after 120 hours of blue light irradiation at 460 nm. For trypan blue, the 1 mm absorbance at 580 nm (specific for trypan blue) decreased from 2.274 ± 0.011 to 1.927 ± 0.045 (r = -0.988, p < 0.001); and for the trypan blue—lutein/zeaxanthin mixture, the 1 mm absorbance at 580 nm decreased from 1.849 ± 0.013 to 0.841 ± 0.000 (r = -0.959, p < 0.001).

Thermal degradation processes were ruled out by comparing absorption spectra of control samples not subjected to irradiation, which were stored at 4°C or room temperature (22–24°C) for 120 hours, respectively. No significant shift of absorption maxima was observed for substance-specific bands at 325, 379, 460, 505 and 580 nm (p > 0.05). With respect to potential photodynamic processes of trypan blue, a linear decline of absorbance was noted during the period of blue-light irradiation. As shown in Fig 2, during 120 hours of irradiating the trypan blue solution, the trypan blue-specific absorbance at 580 nm decreased by 15% from 2.274 ± 0.011 (t = 0 hours) to 1.927 ± 0.045 (t = 120 hours, r = -0.988, p < 0.001); whereas in case of the trypan blue—lutein/zeaxanthin mixture a decrease of absorbance at 580 nm by 55% from 1.849 ± 0.013 (t = 0 hours) to 0.841 ± 0.000 (t = 120 hours, r = -0.959, p < 0.001) was found. The photochemical degradation of trypan blue proceeded 3.67 times faster in the presence of lutein/zeaxanthin. The self-sensitized photodegradation of trypan blue resulted in a decline of the 580 nm absorbance by E_TB580_ = 2.274–0.003 t (t in hours) and concentration by c_TB580_ = 100–0.125 t (c_TB_ in percent). Likewise, the degradation of trypan blue in the presence of lutein/zeaxanthin resulted in a drop of the 580 nm absorption by E_TB580_ = 1.744–0.008 t and concentration by c_TB580_ = 100–0.455 t (c_TB580_ in percent). The absorbance at 379 nm of lutein/zeaxanthin declined by E_L/Z379_ = 4.473–0.016 t (r = -0.821, p < 0.001) and the concentration by c_L/Z 379_ = 100–0.358 t (c_L/Z_ in percent). The extinction of lutein/zeaxanthin diacetate at 460 nm and 505 nm declined similar (r = -0.901, p < 0.001 and -0.912, p < 0.001, respectively). However, due to the spectroscopic overlap of lutein/zeaxanthin and trypan blue the exact estimation of degradation kinetics is limited. Changes of compound-specific absorbance during the process of irradiation are depicted in S2 Fig.

### Degradation products

**Fig 3:**
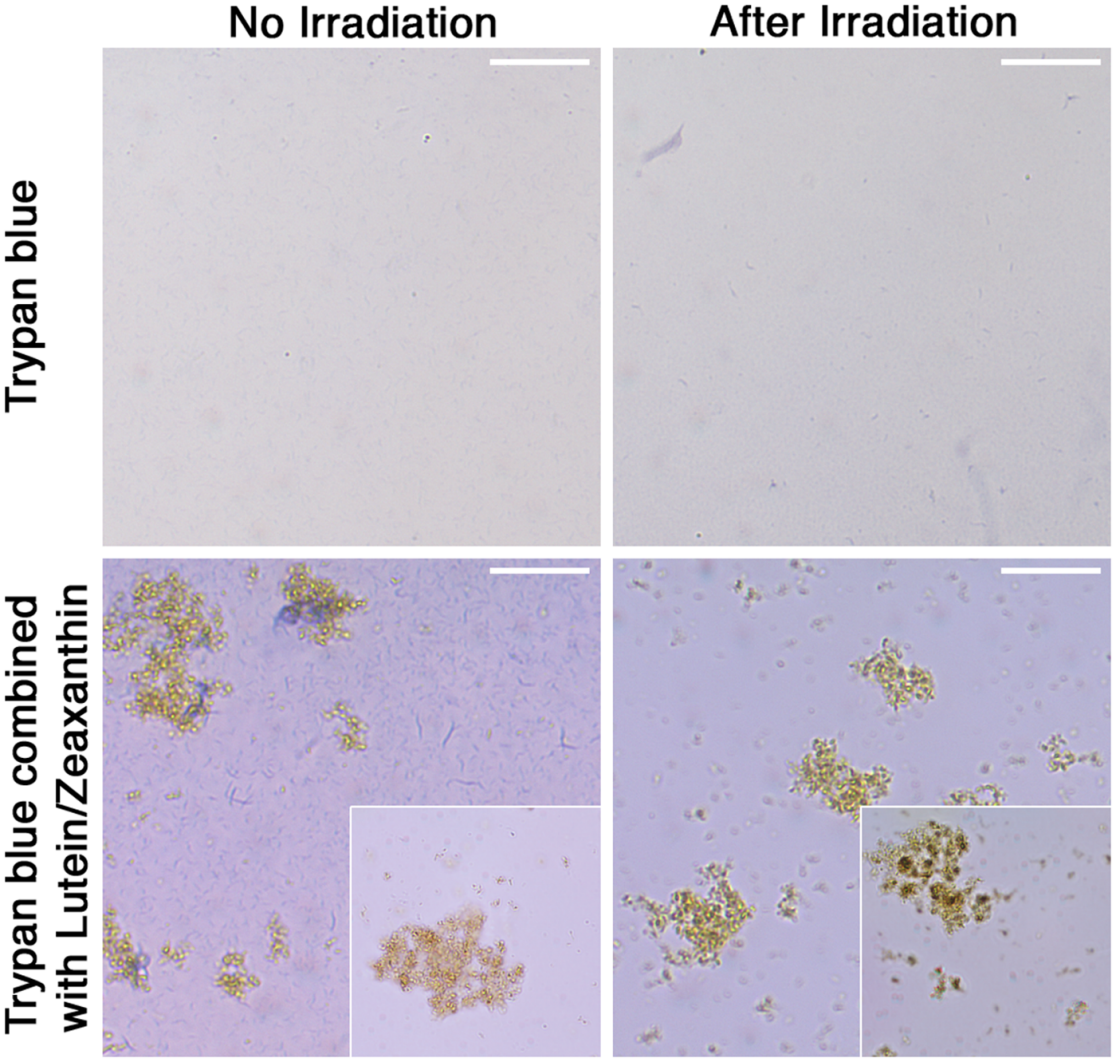
Optical microscopy images of trypan blue and trypan blue combined with lutein/zeaxanthin in aqueous solution with no and after 120 hours of blue-light irradiation at 460 nm. In the trypan blue—lutein/zeaxanthin solution, fine homogenous yellow granules of lutein/zeaxanthin were observed, which changed into heterogeneous brown clots during the process of irradiation, as shown in the viewframe below. Scale bars represent 20 μm.

Dye solutions were examined by high-resolution optical microscopy. While pure trypan blue dissolved homogenously in phosphate-buffered saline and did not present any precipitates before and after irradiation; the mixture of trypan blue and lutein/zeaxanthin revealed microscopically fine homogenous yellow granules of lutein/zeaxanthin, which changed during the irradiation process into heterogeneous brown clots as shown in Fig 3. The precipitates were separated from the soluble part by centrifugation and aliquots for MALDI-TOF mass spectrometry analysis taken from the supernatant solution.

**Fig 4:**
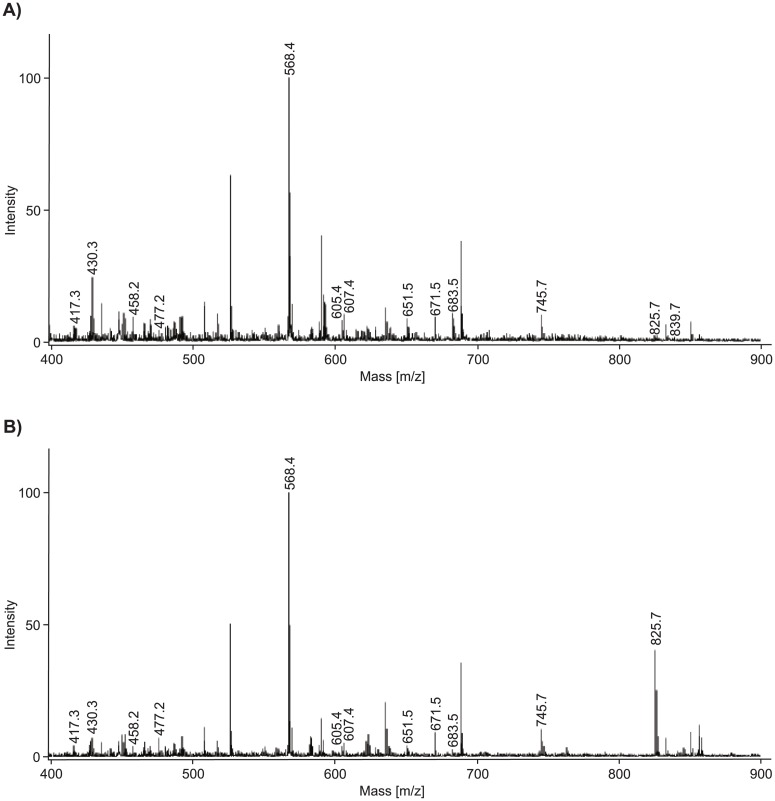
MALDI-TOF spectra of examined dye solutions, comprising of 0.4 mg/mL trypan blue and 10.0 mg/mL lutein/zeaxanthin, with no (a) and after 120 hours of blue light irradiation at 460 nm (b).

**Fig 5:**
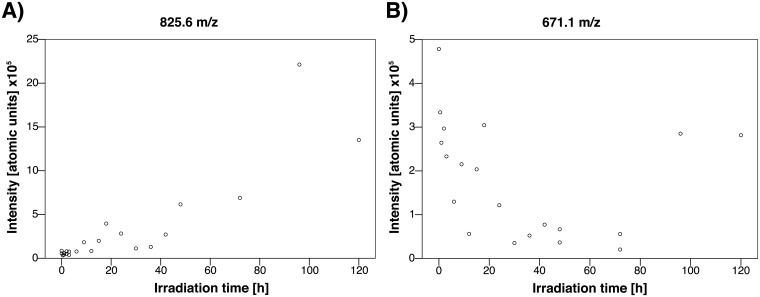
MALDI-TOF mass spectrometry of selected fragment ion peaks for the blue light irradiation series. Distinct mass signatures of trypan blue decomposition intermediates were formed subsequent to dissociation of methoxyamine (m/z = 825.6 [M– 47]^+^) **(a)** that accumulates over time (r = 0.917, p < 0.001); and following dissociation of sulfonyl arin (m/z = 671.1 [M– 47–155]^+^) **(b)** that is consumed in the course of irradiation (r = -0.488, p = 0.040).

**Fig 6:**
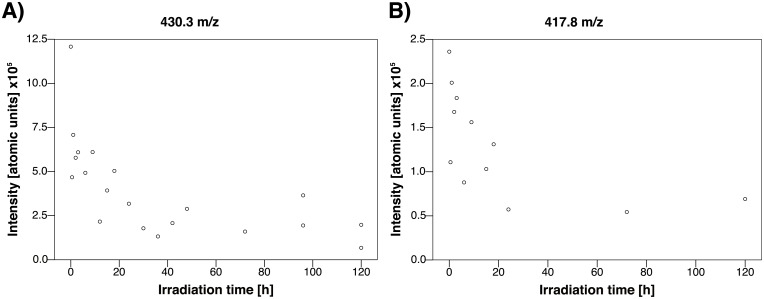
MALDI-TOF mass spectrometry of selected fragment ion peaks during the process of blue light irradiation. Distinct mass signatures of lutein were observed at m/z = 430.3 **(a)** and 417.8 **(b)**, which disappeared in the course of light exposure (r = -0.726, p = 0.005 and r = -0.797, p = 0.002, respectively).

MALDI-TOF mass spectrometry was used to trace substance-specific fragmentation patterns; samples not subjected to irradiation, as shown in Fig 4a, were used. A common fragmentation pattern of lutein/zeaxanthin was observed at m/z = 568.4, 477.1 and 458.9, whereas a distinct lutein signature was found at m/z = 430.3 and 417.8.[33, 34] While trypan blue was detected spectroscopically, its specific fragment ion peak of m/z = 872.9 could not be recorded; most likely due to the complexation by lutein (followed by precipitation) or fragmentation in the MALDI-TOF spectrometer. Analysis of the supernatant revealed distinct trypan blue degradation products identified by fragment ion peaks at m/z = 839.3, 825.6, 745.6, 683.5, 671.1, 651.4 and 607.4. To dispel concerns about potential fragmentations inside the mass spectrometer, fragmentation patterns were tracked during the entire irradiation period of 120 hours (Fig 4b). Intermediates were identified by analyzing mass-differences of characteristic fragment ion peaks within the fragmentation patterns. Their quantity was correlated with the duration of irradiation, as shown in Figs 5 and 6. The degradation of trypan blue might be described as follows: In the first step, methoxyamine (m/z = 825.6 [M– 47]^+^) and dimethyl sulfate (m/z = 745.6 [M– 127.2]^+^) are split off. Significant correlations between the amount of intermediates and the duration of light exposure were found for methoxyamine (methylhydroxylamine, m/z = 825.6, r = 0.917, p < 0.001, Fig 5a). It is proposed that, in a second step, the intermediate species release sulfonyl arin (m/z = 671.1 [M– 47–155]^+^) or phenol (m/z = 651.4 [M– 127.2–94.2]^+^), respectively. Correlations between the amount of intermediates and irradiation time were found for sulfonyl arin (m/z = 671.1: r = -0.488, p = 0.040, Fig 5b) and phenol (m/z = 651.4, r = -0.459, p = 0.055). Further conclusions regarding fragmentations and intermediates remain uncertain due an increased number of low-mass decompositions products formed.

During the irradiation process, a decrease of lutein specific signatures at m/z = 430.3 (r = -0.726, p = 0.005, Fig 6a) and m/z = 417.8 (r = -0.797, p = 0.002, Fig 6b) was observed, which indicates a consumption of lutein.

**Fig 7:**
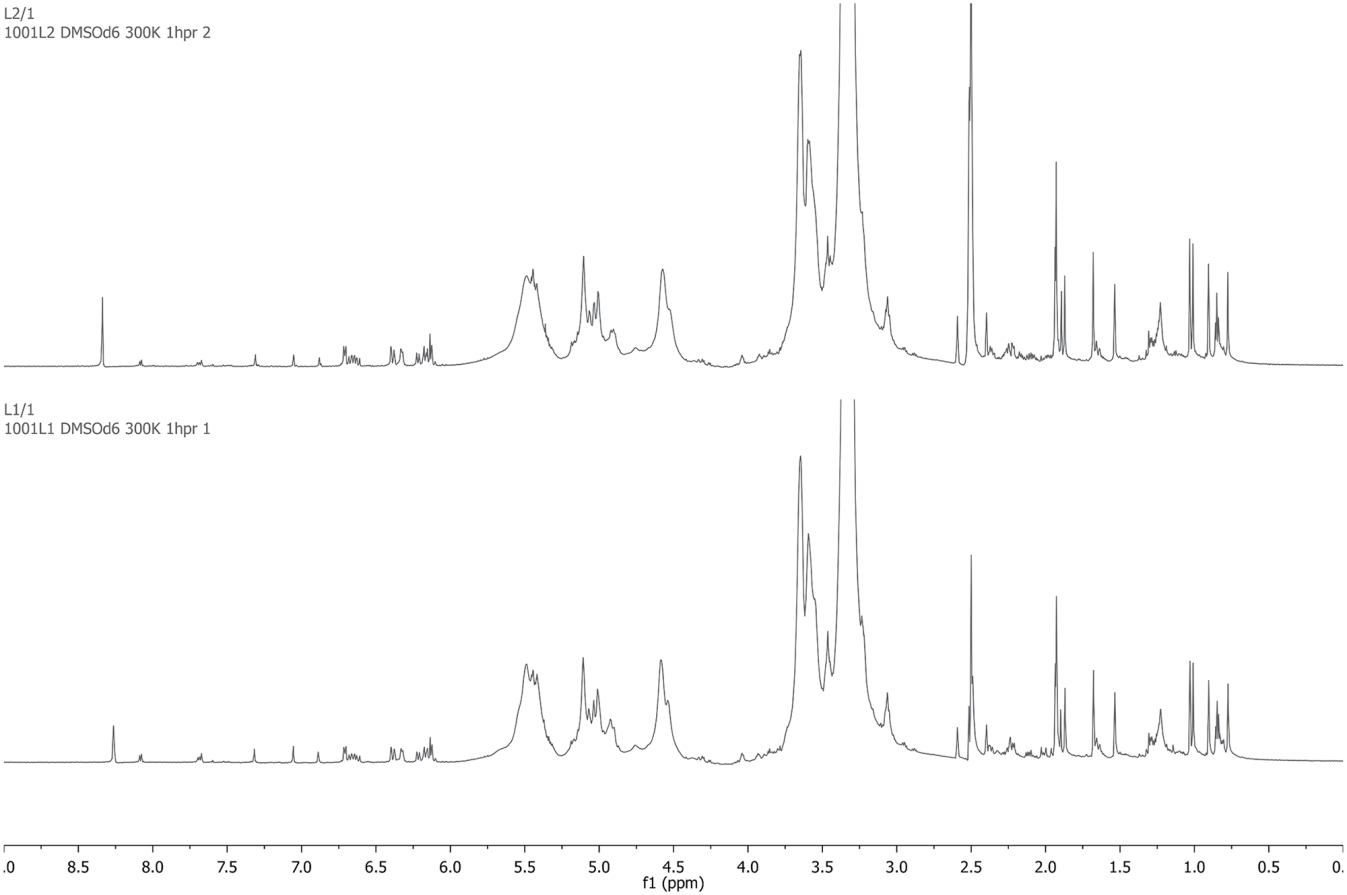
Nuclear magnetic resonance spectra of trypan blue—Lutein/zeaxanthin mixture with no (lower graph) and after 120 hours (top graph) of blue light irradiation at 460 nm. Trypan blue and Lutein/zeaxanthin signals: ^1^H NMR (700 MHz, DMSO-*d*_6_, RT, ppm) *δ* = 8.08 (d, *J* = 8.4 Hz, 2H), 7.69 (d, *J* = 8.4 Hz, 1H), 7.68 (s, 2H), 7.32 (s, 2H), 7.06 (d, *J* = 1.5 Hz, 2H), 6.89 (d, *J* = 1.5 Hz, 2H), *δ* = 6.71 (d, *J* = 8.8 Hz, 2H), 6.69–6.60 (m, 2H), 6.39 (d, *J* = 14.9 Hz, 2H), 6.33 (d, *J* = 8.8 Hz, 2H), 6.22 (d, *J* = 11.5 Hz, 2H), 6.19–6.09 (m, 5H) ppm.

**Fig 8:**
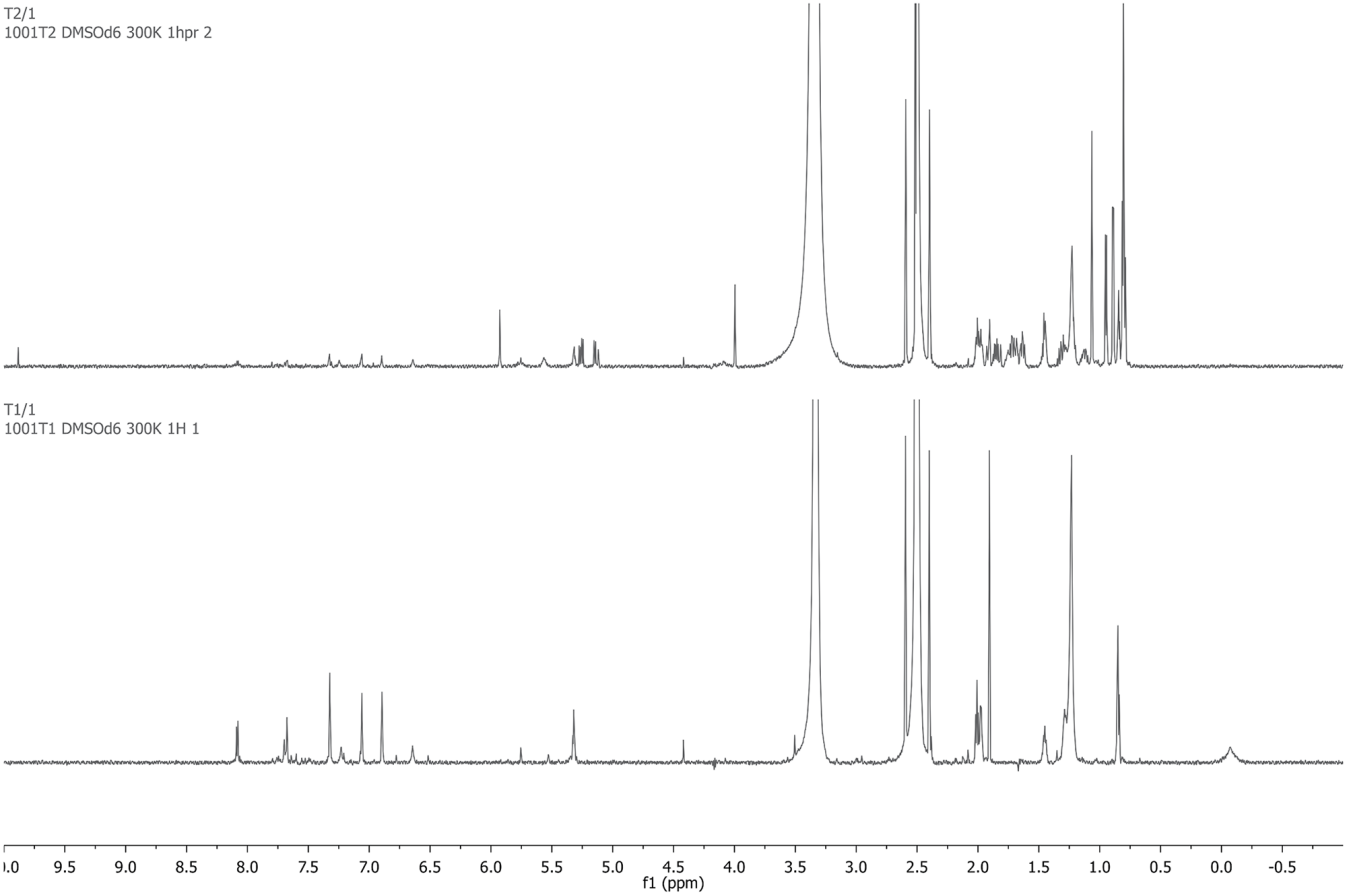
Nuclear magnetic resonance spectra of trypan blue with no (lower graph) and after 120 hours (top graph) of blue light irradiation at 460 nm. Trypan blue signals: ^1^H NMR (700 MHz, DMSO-*d*_6_, RT, ppm) *δ* = 8.08 (d, *J* = 8.4 Hz, 2H), 7.69 (d, *J* = 8.4 Hz, 1H), 7.68 (s, 2H), 7.32 (s, 2H), 7.06 (d, *J* = 1.5 Hz, 2H), 6.89 (d, *J* = 1.5 Hz, 2H) ppm. Dimethyl sulfate signal: ^1^H NMR (700 MHz, DMSO-*d*_6_) *δ* = 4.00 ppm (top graph).

**Fig 9:**
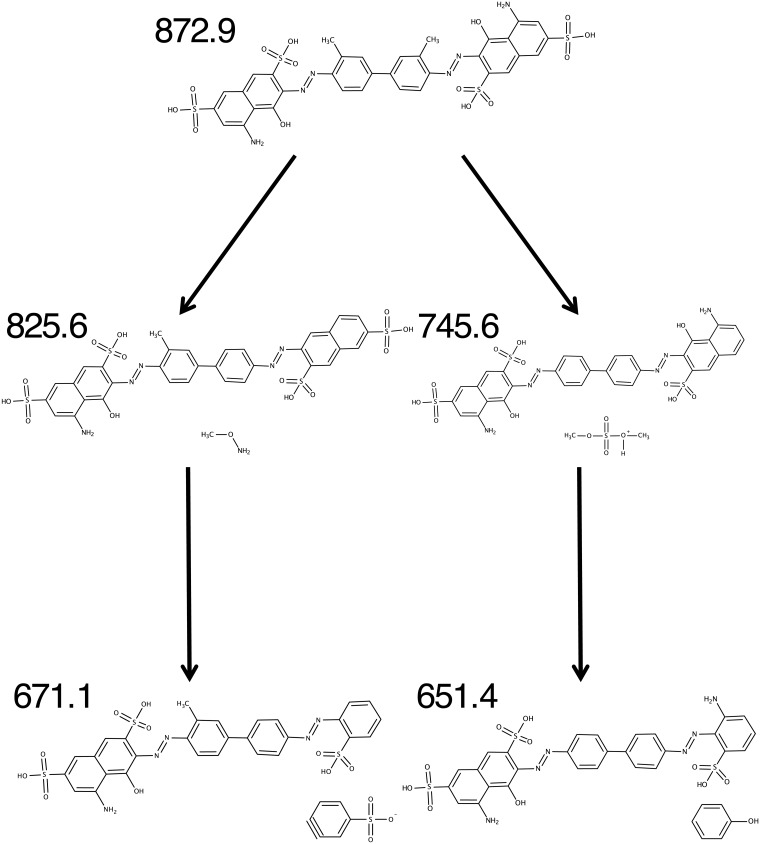
Schematic illustration of proposed major/preferred trypan blue photochemical degradation pathways (primary and secondary steps, respectively). It is suggested that the self-sensitized photodegradation of trypan blue occurs under dissociation of dimethyl sulfate (m/z = 745.6 [M– 127.2]^+^), presumably followed by elimination of phenol (m/z = 651.4 [M– 127.2–94.2]^+^). In the presence of lutein/zeaxanthin, photochemical degradation of trypan blue is triggered and performs under presumed generation of methoxyamine (m/z 825.6 [M– 47]^+^), followed by dissociation of sulfonyl arin (m/z = 671.1 [M– 47–155]^+^).

Nuclear magnetic resonance spectrometry was used for verification. Trypan blue signals were detected as shown in Figs 7 and 8: ^1^H NMR (700 MHz, DMSO-*d*_6_) *δ* = 8.08 (d, *J* = 8.4 Hz, 2H), 7.69 (d, *J* = 8.4 Hz, 1H), 7.68 (s, 2H), 7.32 (s, 2H), 7.06 (d, *J* = 1.5 Hz, 2H), 6.89 (d, *J* = 1.5 Hz, 2H) ppm. Signals from -OH, -SO_3_H and -NH_2_ functionalities were not detected due to interactions with residual water; signals from -CH_3_ groups were obscured by DMSO signals. Lutein/zeaxanthin signals are shown in Fig 7: ^1^H NMR (700 MHz, DMSO-*d*_6_) *δ* = 6.71 (d, *J* = 8.8 Hz, 2H), 6.69–6.60 (m, 2H), 6.39 (d, *J* = 14.9 Hz, 2H), 6.33 (d, *J* = 8.8 Hz, 2H), 6.22 (d, *J* = 11.5 Hz, 2H), 6.19–6.09 (m, 5 H) ppm; signals from -CH_3_, -CH_2_-, and -CHOH were obscured or unassignable. Methoxyamine is a colorless volatile liquid in polar solvents, which quickly decomposes into methane and nitroxyl and could not be detected by NMR. Yet, dimethyl sulfate was detected by its signal at *δ* = 4.00 ppm after trypan blue irradiation,[35] as shown in Fig 8. In summary, relevant proposed reaction steps are illustrated in Fig 9.

## Discussion

So far, the intraocular application of trypan blue has been considered relatively safe within the anterior segment,[9] while clinical and experimental investigations on retinal tissues demonstrated a dose-, time- and light-dependent toxicity.[13, 14, 18–20, 23] Self-sensitized photodynamic properties have extensively been investigated for indocyanine green,[36–38] whereas mechanisms and circumstances of the retinal toxicity of trypan blue remained uncertain. In view of diverse toxicity profiles of trypan blue within the anterior and posterior ocular segment, we decided to study interactions of trypan blue with lutein/zeaxanthin to contribute to a better understanding of the underlying degradation processes.

In the following, we will focus on reliably evidenced degradation metabolites and mechanism to reduce the room for speculations. While the degradation is quite complex for the involved solutions, formulations and processes, we like to emphasize that the photochemical degradation is also accompanied by the formation of volatile substances and reactive oxygen species, which are not explicitly analyzed hereinafter.

Our results demonstrate a slight but continuous self-degradation of trypan blue under blue-light irradiation. Decomposition products were concluded from mass spectrometric analyses, which indicate the formation of methoxyamine (methylhydroxylamine), dimethyl sulfate, phenol and sulfonyl arin while the preferred degradation pathway depends on the presence of lutein/zeaxanthin. The self-sensitized photodegradation of trypan blue occurs under generation of dimethyl sulfate and presumed formation of phenol. In contrast, results of trypan blue decomposition in the presence of lutein/zeaxanthin indicate the formation of methoxyamine and sulfonyl arin. In this context, the self-sensitized photodegradation of lutein/zeaxanthin is well understood, and does not generate methoxyamine and sulfonyl arin by itself.[39] Photodegradation of trypan blue proceeds almost four times faster in the presence of lutein/zeaxanthin, compared to the self-sensitized reaction. Cytotoxicity is known for hydroxylamine[40, 41] and dimethyl sulfate[42, 43]; whereas sulfonyl arin is a highly reactive intermediate which is suspected to induce cellular damage.[44]

The mechanism of lutein-triggered photochemical degradation of trypan blue is of clinical relevance, primarily because of its widespread use as intraocular surgical adjuvant. Corresponding to our findings, the blue-light degradation of the widespread commercial dye crystal violet in the presence of the antioxidant riboflavin has also been recently revealed under the formation of reactive oxygen species.[45] While these kind of mechanisms might be used for aimed industrial photocatalysis;[46–48] in vivo, the above-mentioned metabolites are potential hazards.

In 2008 Engel et al. were able to disclose mechanisms of indocyanine green self-decomposition by singlet oxygen under light exposure.[38] Their findings led to a better understanding of what might cause retinal damages after indocyanine green-assisted macular surgery;[49] and also initiated a re-evaluation of its’ application during chromovitrectomy.

As previously discussed, trypan blue-associated toxicity might be reduced by lowering the intraocular dosage and the duration of tissue-exposure. However, potential risks are not limited to the time of surgery, but may also emerge postoperative from residues of trypan blue on ocular tissues. Trypan blue may last intraocular for hours to days, and can diffuse form the anterior to the posterior segment.[20] Even though the process of photochemical degradation is slow; our data has shown that ten percent of the starting materials have decomposed into toxic products within 24 hours.

In conclusion, our findings contribute to the understanding of trypan blue photodegradation, which can be triggered by adjuvant substances, such as lutein or zeaxanthin, and results in the generation of cytotoxic products. For this reason, trypan blue should be considered with care and intraocular residues need to be avoided to keep application safety high.

## Supporting information

S1 Fig MALDI-TOF spectrum of a dextran ladder for referencing. (PDF)

S2 Fig Graphs of specific 1 mm absorbance of the trypan blue—Lutein/zeaxanthin mixture at 379 nm (a, lutein/zeaxanthin), 460 nm/505 nm (b and c, lutein/zeaxanthin diacetate) and 580 nm (d, trypan blue) during blue light irradiation at 460 nm. The absorbance decreases as follows: lutein/zeaxanthin (r = -0.821, p < 0.001), trypan blue (r = -0.959, p < 0.001) and lutein/zeaxanthin diacetate (r = -0.901, p < 0.001 and -0.912, p < 0.001, respectively), which indicates that all compounds are decompesed/consumed during the photochemical reaction.
(PDF)

S3 Fig Nuclear magnetic resonance spectra of trypan blue with no (lower graph) and after 120 hours (top graph) of blue light irradiation at 460 nm (raw data). (PDF)

S4 Fig Nuclear magnetic resonance spectra of trypan blue—Lutein/zeaxanthin mixture with no (lower graph) and after 120 hours (top graph) of blue light irradiation at 460 nm (raw data). (PDF)

S1 File UV/Vis spectrometric data of blue light irradiation series of trypan blue and trypan blue lutein/zeaxanthin (SPSS file with raw data). (SAV)

S2 File MALDI-TOF mass spectrometric data of blue light irradiation series of trypan blue and trypan blue lutein/zeaxanthin (SPSS file with raw data). (SAV)
