# Supplementary figures and images for: Photochemical degradation of trypan blue
Source: PLoS One. 2018 Apr 10;13(4):e0195849. doi: 10.1371/journal.pone.0195849 (PMC5892916; doi:10.1371/journal.pone.0195849)

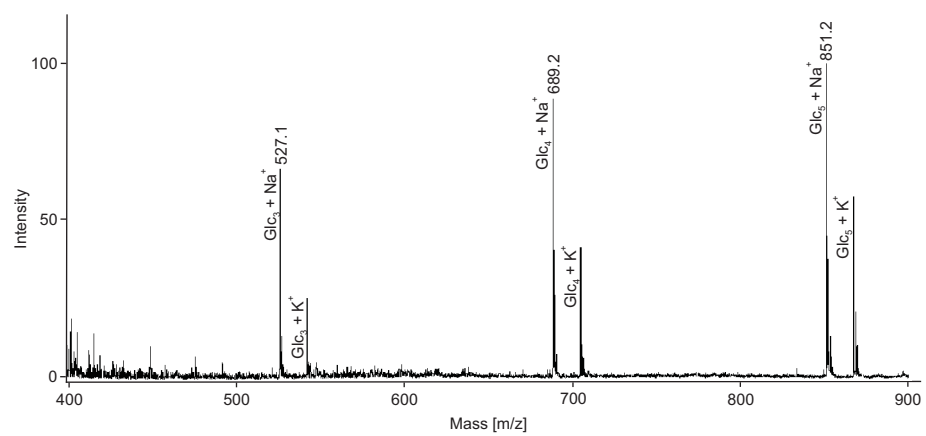

Supplement: S1 Fig — (PDF) [file pone.0195849.s001.pdf]

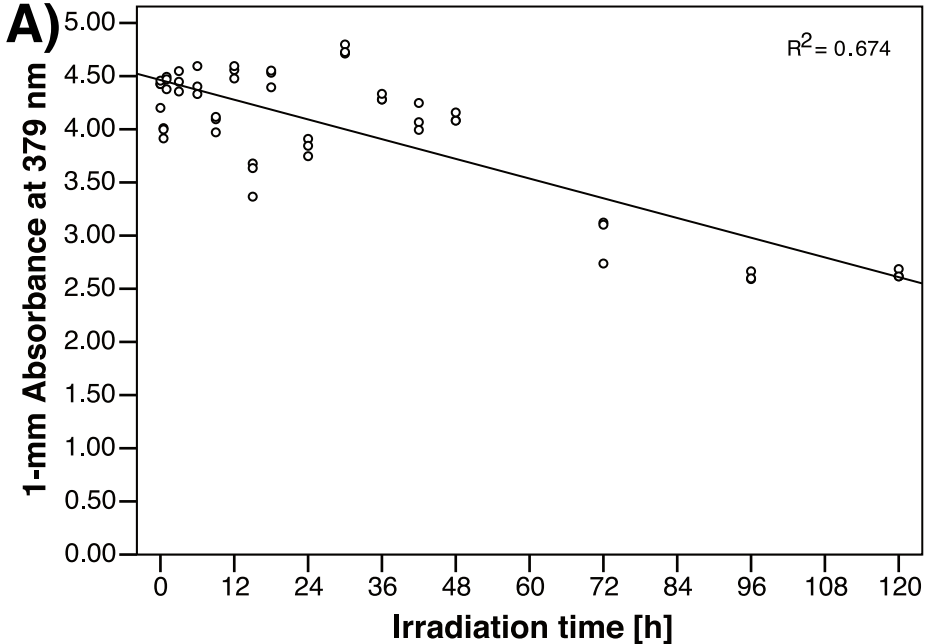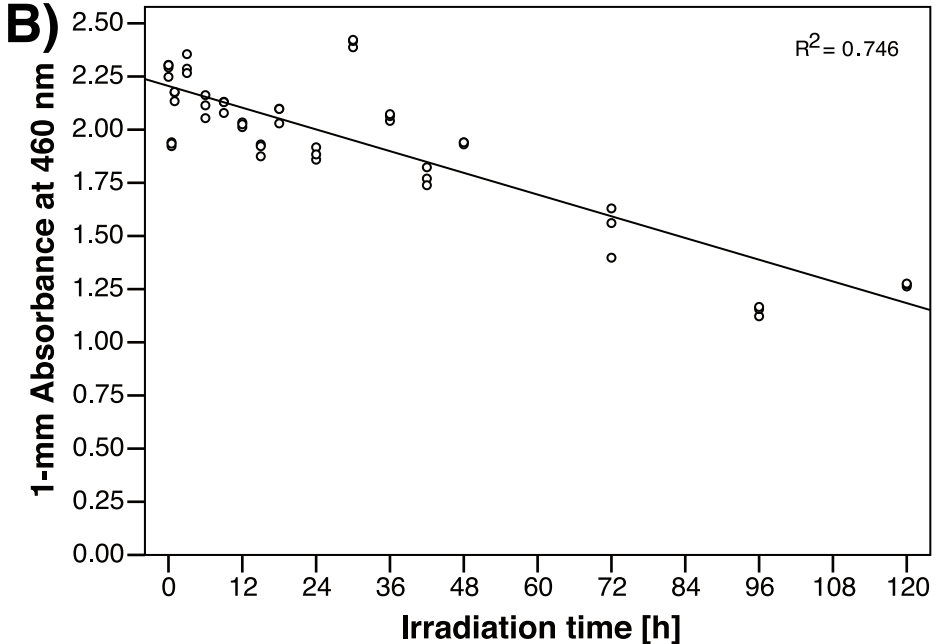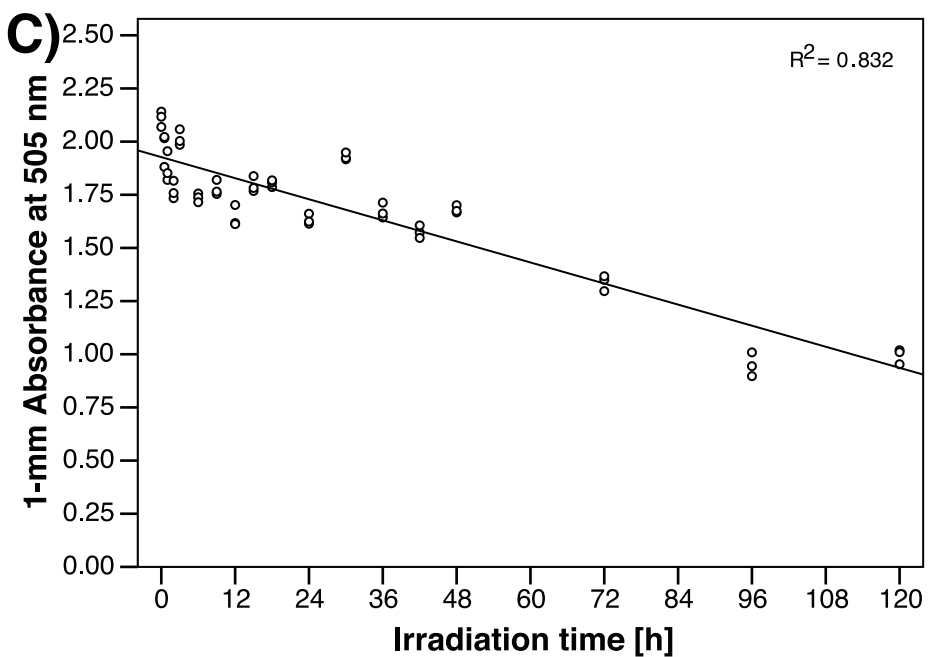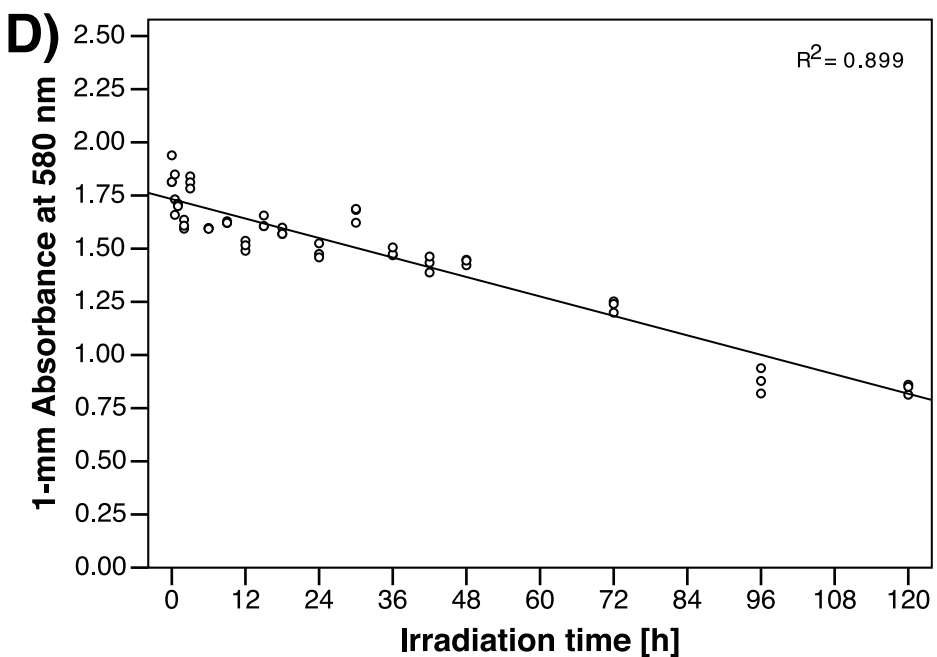

Supplement: S2 Fig — The absorbance decreases as follows: lutein/zeaxanthin (r = -0.821, p < 0.001), trypan blue (r = -0.959, p < 0.001) and lutein/zeaxanthin diacetate (r = -0.901, p < 0.001 and -0.912, p < 0.001, respectively), which indicates that all compounds are decompesed/consumed during the photochemical reaction. (PDF) [file pone.0195849.s002.pdf]

T2/1  
1001T2 DMSOd6 300K 1hpr 2

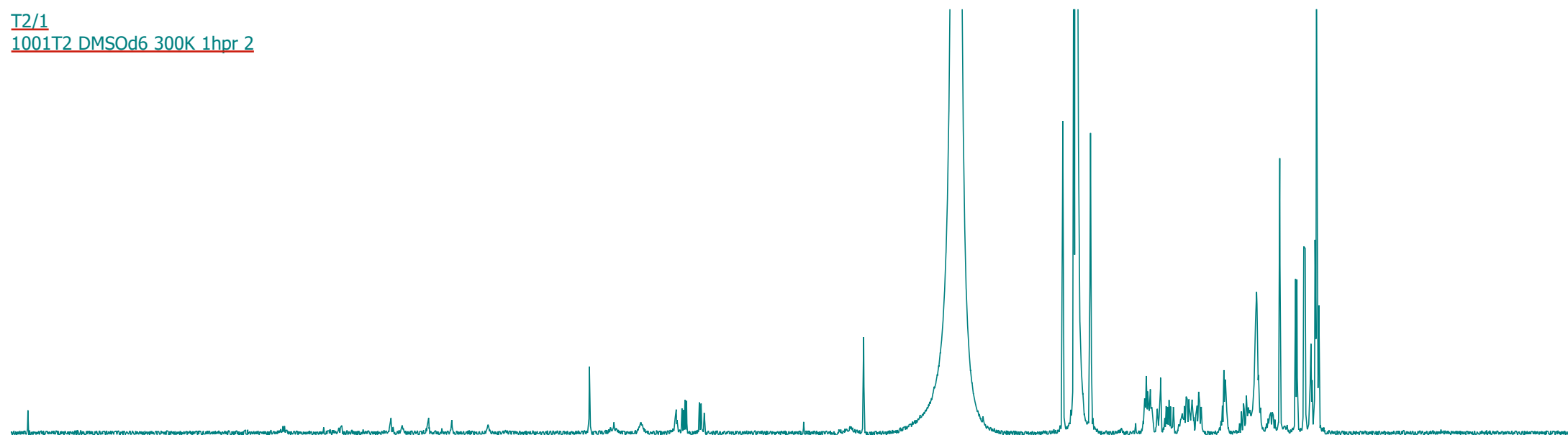

T1/1  
1001T1 DMSOd6 300K 1H 1

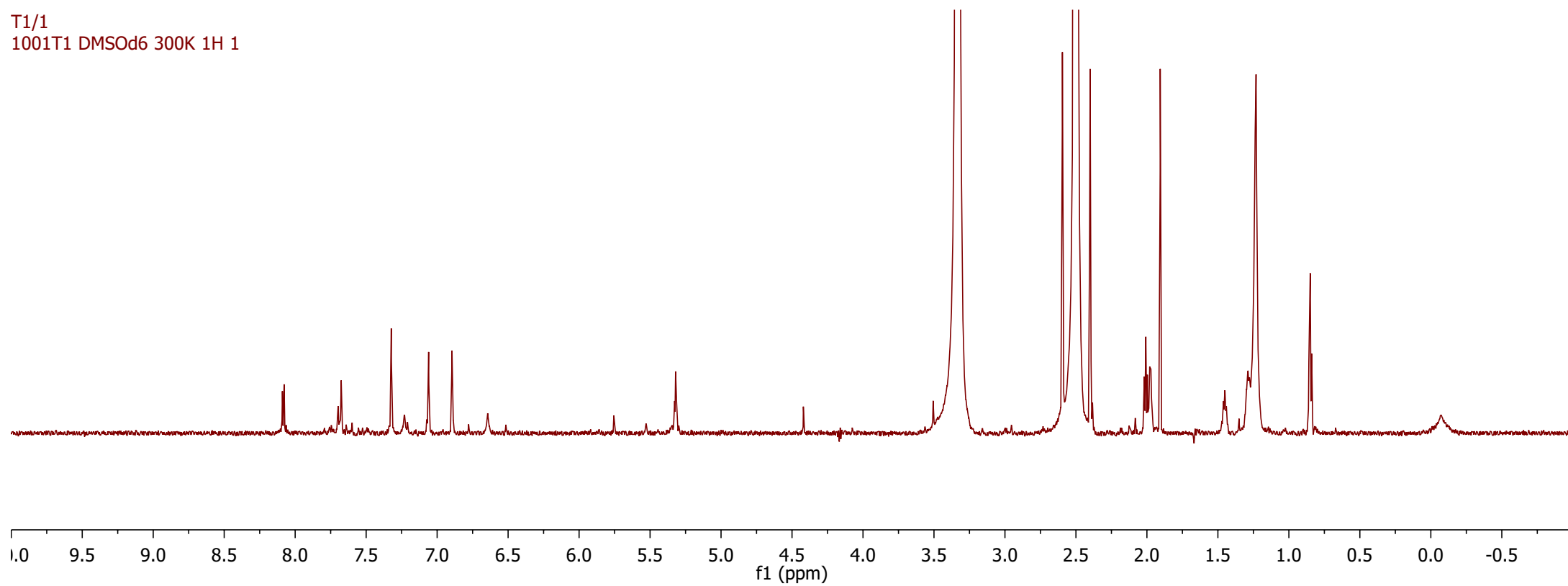

Supplement: S3 Fig — (PDF) [file pone.0195849.s003.pdf]

L2/1  
1001L2 DMSOd6 300K 1hpr 2

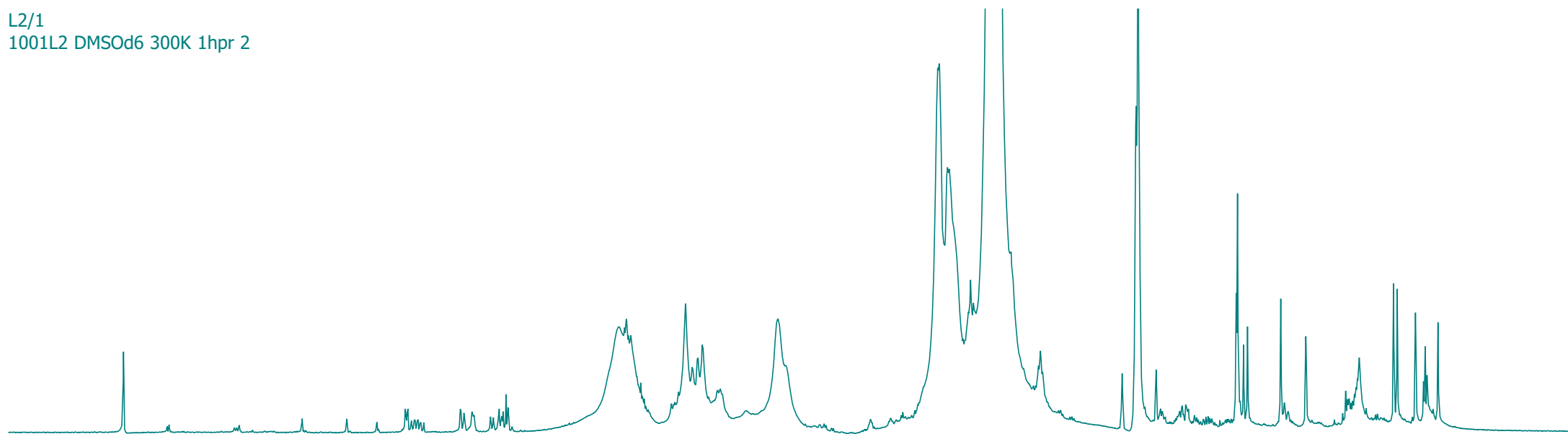

L1/1  
1001L1 DMSOd6 300K 1hpr 1

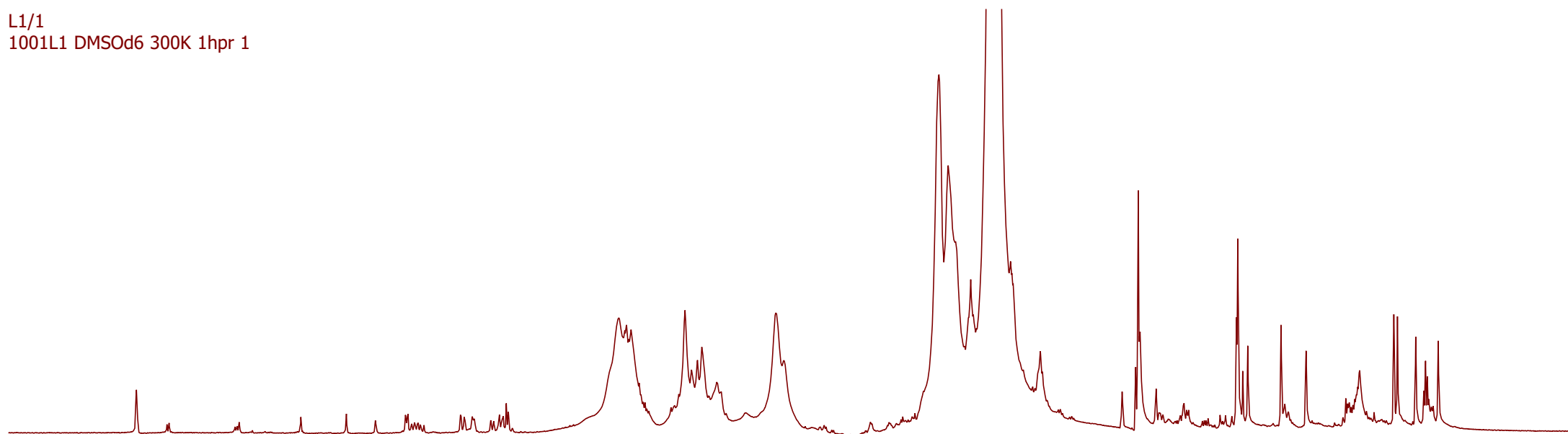

0.0 8.5 8.0 7.5 7.0 6.5 6.0 5.5 5.0 4.5 4.0 3.5 3.0 2.5 2.0 1.5 1.0 0.5 0.0  
f1 (ppm)

Supplement: S4 Fig — (PDF) [file pone.0195849.s004.pdf]
